# Developing a digital heart failure symptom management intervention using the preparation phase of the Multiphase Optimization StraTegy

**DOI:** 10.1186/s12912-026-05571-2

**Published:** 2026-10-22

**Authors:** Michelle van Rijn, Tiny Jaarsma, Gerhard Andersson, Subhash Aryal, Kenneth Freedland, Christopher Lee, Shayleigh Page, Sara Pettersson, Barbara Riegel, Anna Strömberg, Ercole Vellone, Heleen Westland

**Affiliations:** 1 Julius Center for Health Sciences and Primary Care, Department of General Practice and Nursing Science, University Medical Centre Utrecht, Utrecht, The Netherlands; 2 Department of Health, Medicine and Caring Sciences, Linköping University, Linköping, Sweden; 3 Department of Behavioural Sciences and Learning, Linköping University, Linköping, Sweden; 4 HEI-Lab: Digital Human-Environment Interaction Labs, Lusófona University, Lisboa, Portugal; 5 Johns Hopkins University (School of Nursing), Baltimore, USA; 6 Washington University School of Medicine, Saint Louis, USA; 7 Boston College William F. Connell School of Nursing, Chestnut Hill, USA; 8 Hunter-Bellevue School of Nursing, Hunter College of The City University of New York, New York, USA; 9 Division of Nursing, Department of Neurobiology, Care Sciences and Society, Karolinska Institutet, Stockholm, Sweden; 10 Center for Home Care Policy & Research at VNS Health, New York, USA; 11 School of Nursing, University of Pennsylvania, Philadelphia, PA USA; 12 Department of Cardiology, Linköping University, Linköping, Sweden; 13 Tor Vergata University of Rome, Rome, Italy; 14 Wroclaw Medical University, Wroclaw, Poland

**Keywords:** Heart failure, Self-care, Symptom burden, Symptom assessment, Internet-based intervention

## Abstract

**Background:**

Persons with heart failure (HF) often experience bothersome symptoms in daily life, which can negatively affect their health-related quality of life (HRQOL). Self-care e.g. to monitor and manage symptoms, is essential in HF management. Self-care can stabilize the disease, reduce symptoms, and improve outcomes. However, patients often find it difficult to recognize and manage symptoms due to various factors. Consequently, many people delay acting until their symptoms become too severe to manage by themselves. This highlights the need for timely engagement in symptom monitoring and interpretation to enable early and appropriate responses. In the SympTom mAnagement and self-caRe (STAR) study, we prepare (phase 1), optimize (phase 2), and evaluate (phase 3) a digital intervention using the Multiphase Optimization StraTegy (MOST). The STAR intervention supports patients with HF in recognizing their symptoms and taking timely, appropriate actions.

**Objective:**

The aim of this study is to describe the MOST preparation phase of the STAR intervention, including its development of the underlying conceptual model, identification of candidate components and optimization criterion, and assessment of intervention feasibility to inform the subsequent optimization phase.

**Methods:**

We conducted four sequential activities from the preparation phase of MOST: (1) developing a conceptual model; (2) identifying candidate components; (3) identifying optimization criterion; and (4) conducting a feasibility study of the digital STAR intervention with patients with HF and HF nurses.

**Results:**

The conceptual model includes four candidate components; a basic component, symptom awareness training, symptom management counselling, and symptom management experiments. We expect these components to influence behavioral outcomes such as reduced symptom burden and hospitalizations, and improved self-care and HRQOL. The set of candidate components were specified at two levels (low versus high) by varying intensity, frequency, or mode of delivery while maintaining consistent core content. Ten patients with HF and 5 HF nurses tested the STAR intervention in the feasibility study. The STAR intervention was perceived as feasible from both a patient and nurses’ perspective. Patients rated the intervention as clear, user-friendly and structured. Participants valued personal support from HF nurses and perceived it as important for continued use of the intervention. The feasibility findings provide concrete input to refine the intervention prior to the optimization trial.

**Conclusion:**

The STAR intervention is feasible and acceptable to both patients with HF and HF nurses. Combined with the conceptual model, linking components to underlying mechanisms and outcomes, the findings provide us a solid foundation for optimizing the STAR intervention in a subsequent optimization trial.

**Supplementary Information:**

The online version contains supplementary material available at https://doi.org/10.1186/s12912-026-05571-2.

## Introduction

Heart failure (HF) is a progressive and chronic condition that affects approximately 64 million people worldwide [1]. Advances in HF treatment have improved survival rates and as a result, individuals are living longer with symptoms that may negatively affect their quality of life [2]. The daily life of patients can be disrupted by bothersome symptoms such as shortness of breath, fatigue, sleep disturbances, depressive symptoms, and anxiety [2]. To manage symptoms and cope with the chronic and progressive nature of HF, self-care is essential in HF management [3, 4].

Self-care is a set of behavioral process which contain three core concepts: self-care maintenance, self-care monitoring, and self-care management [4]. Self-care maintenance means engaging in behaviors known to maintain health and emotional and physical stability [4]. For example, taking medication as prescribed, eating healthy, staying physically active, and managing stress [4]. Self-care monitoring means continually, attentively observing of how someone feels. This process requires self-awareness and body-listening, which enables patients to notice subtle bodily changes that may indicate a shift in their condition [4]. Self-care management involves responding to signs and symptoms when they occur and acting to restore stability and prevent further deterioration (4). Patients need to evaluate their symptoms, decide if action is needed, and take appropriate actions such as adjusting diuretics, modifying activity levels, or contacting a healthcare provider [4]. Effective self-care can stabilize HF, alleviate symptom burden, improve overall wellbeing, and reduce hospitalizations and mortality [4, 5]. Despite the importance of self-care, patients find it challenging to monitor and manage their symptoms.

Effective self-care requires patients to recognize symptoms, determine if these symptoms indicate worsening HF, and take appropriate actions [4]. This process involves both interoception and symptom attribution [6]. Interoception refers to the ability to perceive and respond to internal bodily signals, such as symptoms [7, 8]. Symptom attribution involves interpreting these symptoms and determining whether they are caused by worsening HF or by other factors [9, 10]. Accurate symptom attribution is important because it guides timely care-seeking and appropriate self-care actions. However, patients may struggle to perceive and interpret bodily signals, which delays or prevents them from recognizing symptom accurately. Even if patients detect symptoms, they may attribute them to normal aging, comorbid conditions, or other causes and some patients may also normalize symptoms by adapting their daily routines to minimize them [11, 12]. Furthermore, 20% of patients with HF experience depressive symptoms, which can blunt self-care by reducing motivation and the capacity to engage in healthy behaviors [3, 4, 13]. As a result, patients often delay self-care actions until symptoms become frequent, bothersome, severe, or persistent or to fade away [14].

Multiple interventions have been developed to strengthen self-care in chronic conditions [15]. However, many of these interventions are complex as they target different behaviors and processes, and are delivered through various modes such as support calls, keeping diaries, and feedback [16]. These interacting components are often delivered simultaneously [16]. Traditionally, intervention development follows a classical approach in which intervention components are selected, sometimes pilot tested, and then combined into a single intervention package that is evaluated in an RCT [17]. If this RCT demonstrates a statistically and clinically significant effect, the intervention is considered effective and ready for implementation and dissemination in routine practice. While this approach establishes overall effectiveness, it provides limited insight into which individual components of the intervention, at what levels, and in what combinations, contribute most to the outcomes [18, 19]. Thus, other efficient research strategies are needed to determine the optimal intervention package [17], such as the Multiphase Optimization Strategy (MOST).

### The Multiphase Optimization Strategy (MOST)

The Multiphase Optimization Strategy (MOST) provides a suitable strategy to unravel the effectiveness of complex interventions [19, 20]. MOST is a structured framework for developing, optimizing, and evaluating complex interventions before dissemination in routine care. The central principle of MOST is that individual components of interventions should first be systematically optimized before the effectiveness of the intervention is tested in an RCT [17]. MOST consists of three phases: the preparation phase, optimization phase, and the evaluation phase. In the preparation phase (phase 1), the groundwork for the optimization and evaluation phases is set [17]. During this phase, a conceptual model is developed, and candidate intervention components and optimization criteria are identified. Also, a feasibility or a pilot study is conducted. In the optimization phase (phase 2), the goal is to build an optimized intervention [17]. Optimization of the intervention is achieved through an optimization trial, such as a factorial experiment, which depends on the nature of the intervention [17]. The optimization trial provides information on which components and component levels contribute most to the outcomes. A component level represents a variation of the candidate component to be examined in the trial. Generally, two levels such as ‘on versus off’ or ‘low versus high’ are selected for each component [17]. One version of the component is more basic compared to the other version, and can, for example, differ in mode, number, or duration of sessions. If both versions are equally promising, the more basic version will be included to promote efficiency and affordability of the intervention. Based on the results of the trial, a final set of components is selected to form the optimized intervention. As a result, the optimized intervention includes only those components that make a meaningful contribution to the predefined outcomes.

In the evaluation phase (phase 3), the goal is to confirm effectiveness of the optimized intervention [17]. The effectiveness of the optimized intervention is tested in an RCT, which remains the gold standard in determining the effectiveness of an intervention [19–21]. The RCT compares the effect of an optimized intervention with a suitable control or comparison condition. At this stage, the contribution of individual components is already understood, allowing for a more targeted and informative evaluation [17]. Although RCTs remain resource-intensive, evaluating an optimized intervention represents a more responsible use of these resources than testing an intervention package composed of components that have only been pilot tested. If the RCT confirms a statistically significant and clinically meaningful effect, the optimized intervention can then be disseminated and scaled up. MOST does not replace the RCT [17]. Rather, MOST integrates the RCT within a broader framework to systematically investigate which components, levels, and combinations, lead to the best outcomes. In doing so, MOST aims to develop interventions that are effective, but also affordable, scalable and efficient (EASE) [17].

### The STAR intervention

In line with the rapid growth of eHealth within healthcare, digital interventions increasingly play a central role in the delivery of chronic care [22]. In the STAR study, we develop, optimize, and evaluate a digital intervention using MOST designed to support patients with HF recognize their symptoms and take timely, appropriate actions. The intervention focuses on patients with stable HF who experience daily but not acute symptoms, with the aim to reduce bothersome symptoms and hospitalizations, while improving self-care and quality of life. STAR complements existing HF care by strengthening patients’ ability to recognize, interpret, and appropriately respond to these symptoms independently.

In this paper, we describe the preparation phase of the STAR intervention using MOST. Specifically, we describe how we (1) systematically developed the conceptual model underlying the STAR intervention, (2) identified candidate intervention components, (3) the optimization criterion, and (4) assessed the feasibility of the STAR intervention to inform the subsequent optimization phase.

## Methods

**Fig. 1:**
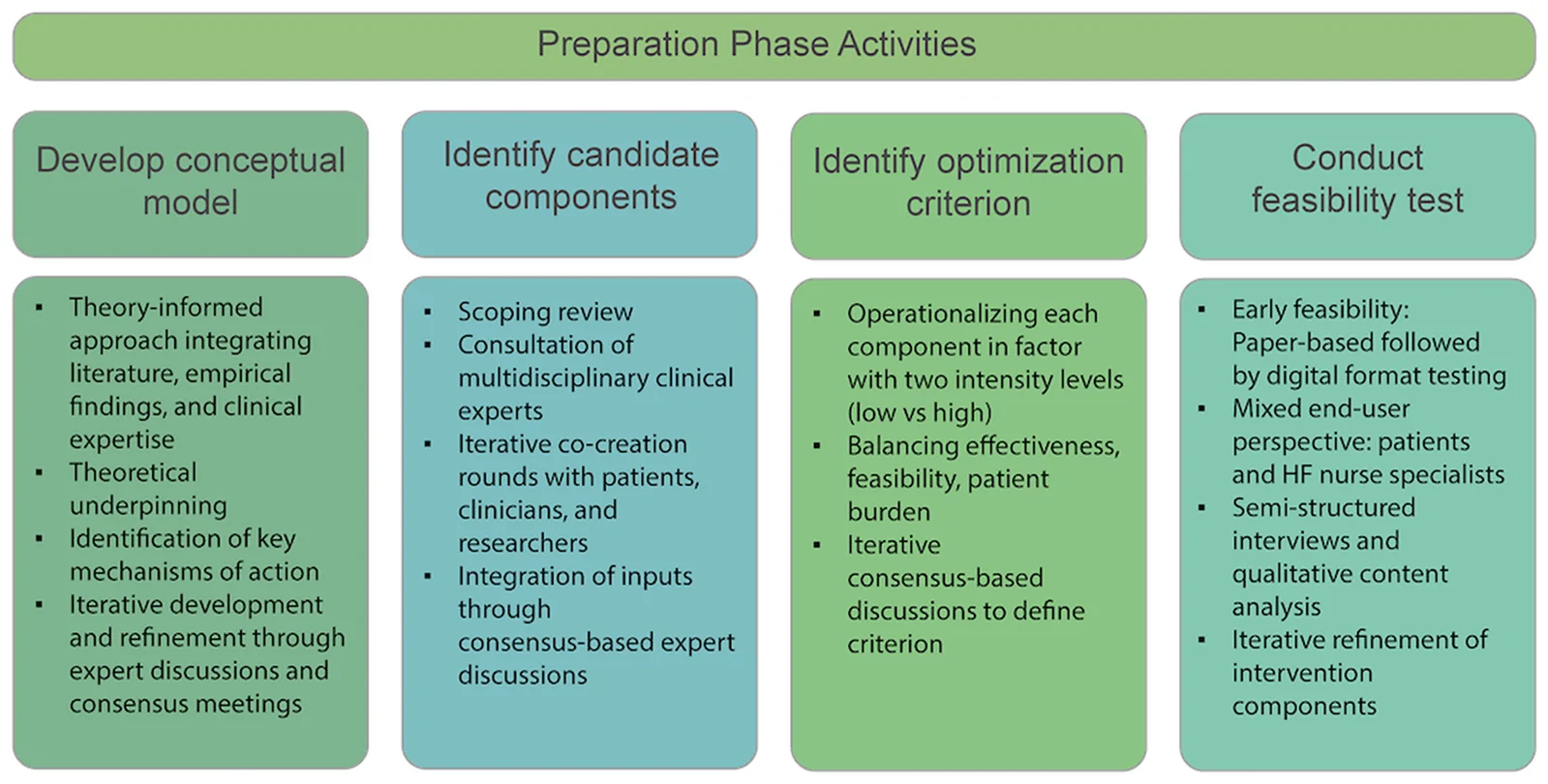
The four activities in the preparation phase of MOST. Each activity shows the steps the research team took within in each activity to prepare the STAR intervention

In the preparation phase (phase 1) of the STAR intervention we conducted four sequential activities: (1) developing a conceptual model; (2) identifying candidate components; (3) identifying optimization criterion; and (4) conducting a feasibility study (Fig. 1) [17]. Although these activities are described sequentially, activities two and three were conducted in parallel. Identifying candidate components and defining optimization criterion are closely related activities, as the optimization criterion determined how candidate components are evaluated and selected for inclusion in the optimized intervention. We describe the development of the STAR intervention according to these four activities. The MOST PREParation REPorting checklist is used to strengthen rigor and reproducibility [23].

### Activity 1. Developing a conceptual model

The conceptual model provides a strong theoretical basis for the development of the STAR intervention. The model helps to guide the selection of candidate intervention components and outcome variables in the next preparation step [17]. We used a theory-informed approach, integrating evidence from the literature with empirical and clinical insights. The conceptual model was underpinned by the Middle Range Theory of Self-Care and Chronic Illness [4, 5]. This theory conceptualizes self-care maintenance, self-care monitoring, and self-care management as related but separate behavioral processes and highlights the central role of symptoms in these processes. The conceptual model was developed by the research team who have been studying self-care symptom monitoring and symptom management processes, and self-care interventions for decades, both as individual researchers and as a team. During multiple iterative meetings, we developed and refined the conceptual model. Empirical and theoretical insights and clinical relevance informed the identification of relevant mechanisms of action towards improved patient short- and long-term outcomes that should be targeted by the STAR intervention.

### Activity 2 and 3. Identifying candidate components and identify optimization criterion

Parallel to the development of the conceptual model, we identified candidate intervention components and defined the optimization criterion to guide the optimization phase of the STAR intervention [17]. Candidate components were selected by the research team to target each of the mechanisms of action described in the conceptual model. Selection was guided by empirical and theoretical insights, clinical relevance and feasibility for use in clinical care. We used the following sources of input: first, we conducted a scoping review of interventions designed to improve interoception and symptom attribution in patients with HF [6]. We mapped existing interventions to identify approaches that support interoception and attribution in these patients. The results of the scoping review contributed to the development and content of the candidate components. Second, we consulted clinical experts, including psychologists, a physiotherapist, a general practitioner, and patient research partners. This ensured the clinical relevance, feasibility, and acceptability of the components and component levels in clinical practice. Third, we held iterative co-creation rounds with patients with HF, HF nurse specialists, cardiologists, and researchers with expertise in self-care. These rounds were held online, lasted between 1 and 1,5 h, and fieldnotes were made. During these rounds, we discussed and refined the proposed components and intensity levels, focusing on relevance, clarity, wording, and patient burden. Thereby ensuring alignment with patients’ needs and preferences. Fourth, our research group integrated these inputs through an iterative consensus-based process. This resulted in a final set of candidate components, each specified at two levels (low versus high) by varying intensity, frequency, or mode of delivery while maintaining consistent core content.

In parallel to identifying the candidate components, we defined the optimization criterion, specifying what is considered the ‘best’ version of the STAR intervention. We defined ‘best’ as achieving improvement in HF symptom monitoring and management in terms of interference of bothersome symptoms, self-care, HF-related QoL, and healthcare utilization. We defined the optimization criterion through a series of discussions within our self-care consortium. These discussions were informed by theory, previous research, the conceptual model and the identified candidate components. Attention was given to balancing intervention effectiveness with patient burden and feasibility of delivery within routine clinical care.

### Activity 4. Conducting a feasibility test

The early-stage feasibility study evaluated the candidate components of the STAR intervention across five of Bowen’s key areas of feasibility: acceptability, demand, implementation, practicality and integration [24]. Bowen defines these areas as: acceptability, the extent to which participants and those delivering the intervention consider it is appropriate, satisfactory, or attractive; demand, the extent users will likely want to use the intervention and the degree of expressed interest; implementation, the extent researchers can successfully deliver the intervention to participants as intended in a real-world setting; practicality, the extent to which the intervention can be carried out using available time, resources, and existing conditions; and integration, the extent to which the intervention can be integrated into existing systems [24]. At this early-stage, we provided the intervention in a paper-based version and then as a digital version on the Iterapi platform [25]. The paper-based version was tested by patients with HF, HF nurse specialists, and a health literacy expert from our advisory board. After reading and testing the material, interviews were held and audio recorded and memos were made. These interviews were conducted by HW and MR. HW is an assistant professor, and MR is a PhD candidate, both with a background in cardiac nursing and self-care research and experienced in both quantitative and qualitative research. HW and MR have a professional relationship with these participants as they are all members of our advisory board. MR and HW processed the results of these first interviews and testing of the paper-based version and refined the digital version and topic list.

For the digital version in Iterapi, both patients with HF and HF nurse specialists were recruited as they both represent end-users for the STAR intervention. Patients were recruited through a Dutch patient organization using a study flyer that outlined the inclusion criteria. Interested patients were able to contact the researchers for participation in the feasibility test and the subsequent interview. Due to insufficient response to the flyer, nurse specialists within our professional network were asked to identify additional patients who might be interested in testing the digital version. HF nurse specialists were recruited using our professional network.

Patients were eligible if they were diagnosed with HF and experiencing symptoms daily, aged ≥18 years, able to read Dutch, and willing to participate in a semi-structured interview. HF nurse specialists were eligible if they work in a HF outpatient clinic and regularly support patients with HF as part of their usual clinical practice. Eligible participants received written and verbal information and gave informed consent before participation. Participants reviewed the digital version at home over a two-week period. We then conducted semi-structured interviews using an interview guide. The interview guide was developed based on the five feasibility domains (acceptability, demand, implementation, practicality and integration) of Bowen with open questions for each domain [24]. We also added questions about participants’ experiences, usability, and preferences regarding the layout of the digital version. The open semi-structured questions allowed for in-depth follow-up questions. The interview guide was reviewed by the study team (HW, TJ, MR) and was not pilot tested. Interviews lasted approximately 45–60 min and were conducted online (Microsoft Teams) or by telephone, based on participant’s preference. Interviews were conducted by MR. MR had no prior relationship with the patients interviewed and had a pre-existing professional relationship with four of the five nurses interviewed as they were recruited from the professional network of MR and HW. Interviews were audio recorded and transcribed verbatim using Amberscript and verified for accuracy (MR). Transcripts were analyzed using a directed content analysis [26]. MR coded all transcripts, and HW independently checked 20% of the transcripts for coding consistency. MR and HW discussed discrepancies in the coding until consensus was reached. To enhance reflexivity, findings and interpretations were regularly discussed during weekly meetings between MR and HW. Questions that arose regarding coding and interpretation were further discussed during biweekly meetings with TJ, allowing consideration of alternative perspectives throughout the analysis process. The number of participants was determined a priori, with a target of 10 patients with HF and 5 HF nurses. Interviews were conducted to obtain patients’ and nurses’ perspectives on the feasibility of the intervention. Data saturation or information power was not used to determine sample size. Member checking was not performed. NVIVO was used to organize data and Microsoft Excel 365 was used to do the qualitative analysis. Participant characteristics were summarized using descriptive statistics in IBM SPSS Statistics (version 26). Findings informed further refinement of the intervention components and content.

### Ethical considerations

The study was conducted according to the principles of the Declaration of Helsinki and Dutch regulations. The Medical Research Ethics Committee (METC) of the University Medical Center Utrecht waived a full ethical review according to the Medical Research Involving Human Subjects Act (WMO).

Prior to the start of the data collection, written informed consent was obtained.

## Results

### Activity 1. Developing a conceptual model

In our initial conceptual model, we included four components: symptom interoception training, symptom evaluation and attribution training, symptom management counselling, and symptom management experiments. But following iterative refinement, symptom interoception training and symptom evaluation and attribution training were merged into a single component, named symptom awareness training, because of substantial overlap in their objectives and content.

**Fig. 2:**
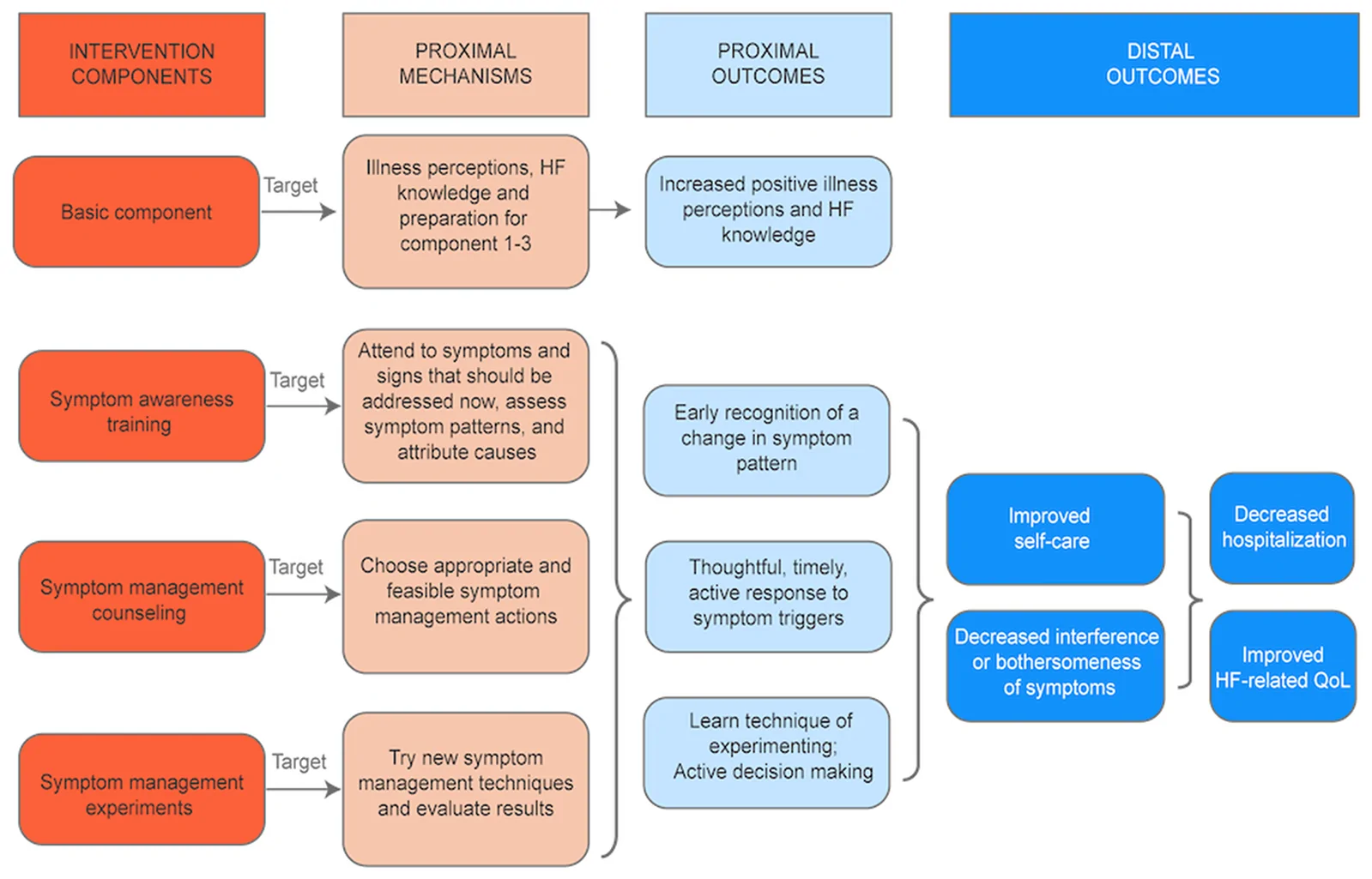
Conceptual model for STAR intervention to improve symptom monitoring and symptom management in patients with HF

The definitive conceptual model specifies the hypothesized pathways through which we expect the STAR intervention improves proximal (short-term) and distal (long-term) outcomes (Fig. 2). The STAR intervention consists of four connected intervention components: (1) a basic component, (2) symptom awareness training, (3) symptom management counselling, and (4) symptom management experiments.

The basic component targets illness perceptions, HF knowledge, and preparation for the subsequent components. The proximal outcome is to strengthen patients’ understanding of HF and to clarify their illness perceptions. Patients often hold unclear or inaccurate beliefs about HF symptoms. These beliefs shape how patients interpret, act upon, and evaluate symptoms, which can delay help-seeking [4, 27]. Strengthening accurate illness beliefs and improving timely action can be achieved with patient education [28, 29].

The symptom awareness training component targets the proximal mechanism of attending to urgent symptoms, assessing symptom patterns, and attributing causes. Symptoms can be vague or even be normalized over time. Consequently, patients misinterpret or underestimate symptoms. This misinterpretation is not due to lack of concern, but because making sense of bodily signals is complex [30]. Paying attention to your body helps people turn symptoms into action [31]. People who are better at noticing and listening to bodily signals are more likely to recognize symptoms as important and respond with appropriate self-care. Such responses include adjusting behavior or seeking help [31]. The proximal outcome is early recognition of changes in symptom patterns because when patients’ awareness is improved, it enables patients to detect condition changes earlier and determine if these changes indicate worsening HF.

The symptom management counselling component targets strengthening patients’ ability to choose appropriate and feasible symptom management actions. Research on HF symptom response demonstrates that patients do not react uniformly [32–34]. Some people recognize symptoms early and act promptly. Others delay, attempt to manage symptoms on their own, or feel uncertain about the appropriate action [32–34]. The proximal outcome is a thoughtful, timely, and active response to symptoms.

The symptom management experiment component encourages patients to try new symptom management techniques and evaluate the results. Its proximal outcome is enactment of behavioral experimentation and active decision making. We hypothesize that proximal outcomes will lead to beneficial distal outcomes. These outcomes include reduced interference of bothersome symptoms, improved self-care, fewer hospital admissions, and improved HF-related quality of life.

### Activity 2 and 3. Identifying candidate components and the optimization criterion

The basic component provides knowledge and supports development of illness perceptions. Therefore, we consider it a necessary component for all participants and will not examine it experimentally during the subsequent optimization phase. The remaining three key components were identified as candidate components to be tested experimentally.

The symptom awareness training component includes exercises that help patients pay attention to bodily signals. The symptom management counselling component uses a structured self-assessment [33]. The symptom management experiments component explains symptom management actions. It encourages patients to test these actions daily and log their experiences.

For each candidate key component, we developed two versions: a low-intensity and a high-intensity version. These versions allow us to examine the effects of the optimal intervention dose and level of nurse support during the optimization trial. The core content of the two versions is the same but differ in the number of exercises and/or the mode and frequency of nurse support. Only the key components are nurse-supported. The number of exercises, mode and frequency of counselling differences were determined through consensus within the research team, informed by expert meetings and patient input. The selected levels were designed to create a clear and meaningful contrast in intervention intensity while remaining acceptable and feasible for both patients and nurses [17].

For the symptom awareness training component, the low-intensity version includes doing the exercises twice within one week and written counselling at the end of the component. The high-intensity version includes doing the exercises six times over two weeks with verbal counselling at the end of the component. For the symptom management counselling component, the low-intensity version includes one counselling session at the end of the component. The high-intensity version includes two counselling sessions: one during and one at the end of the component. For the symptom management experiments component, the low- and high-intensity versions differ in the mode of counselling provided at the end of the component (written versus verbal counselling).

Clinical experts emphasized managing expectations and using positive, normalizing language to introduce information or exercises. They noted that people unfamiliar with mindfulness-based practices may perceive some exercises as abstract. To improve accessibility and acceptability, we replaced technical terms like interoception with familiar language, such as relaxation exercise. Experts advised against including full mindfulness training to improve symptom awareness, because they typically deliver mindfulness as a therapeutic intervention. Instead, we refined the intervention to include brief, concrete mindfulness-based exercises for easier integration in routine care. Finally, experts recommended focusing on a single symptom rather than multiple symptoms in the intervention. We refined the intervention to help patients identify one symptom that was most bothersome in their daily live and manageable to action. We expect this refinement improves clarity, reduce complexity, and increase patients’ control over their symptoms.

**Fig. 3:**
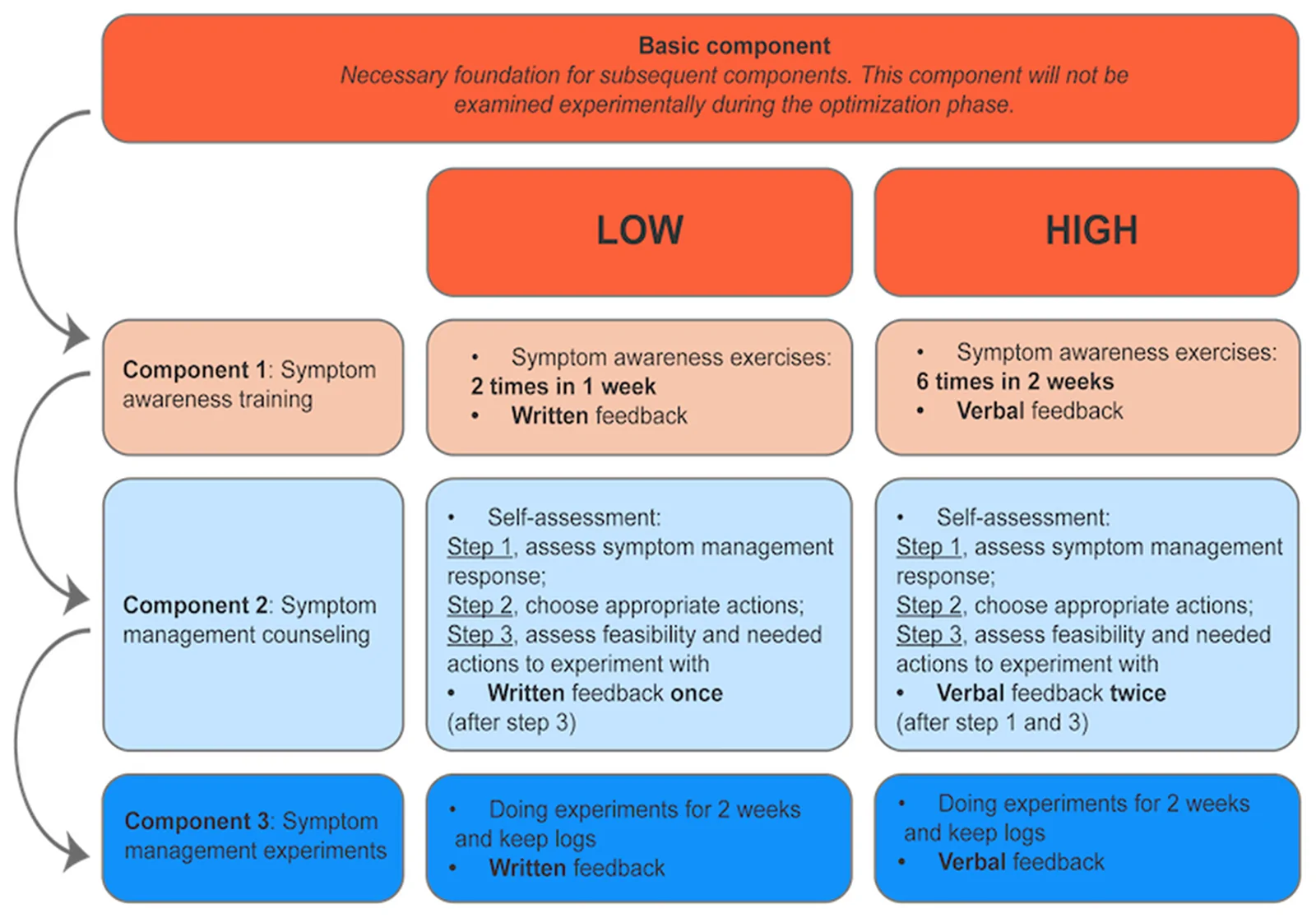
Candidate components with low and high versions of the STAR intervention. The core content of the low- and high-intensity is the same. The number of exercises and/or the mode and frequency of nurse support differs between the two versions

The component levels allow the evaluation of the individual and combined effects of the candidate components during the optimization phase. The optimization criterion for the STAR intervention was defined as selecting the combination of components that is expected to lead to the greatest improvement in symptom related self-care, while remaining feasible to implement in routine HF care. Figure 3 presents an overview of the candidate components and their levels.

### Activity 4. Conducting a feasibility test

Fifteen participants (10 patients, 5 nurses) took part in early-stage feasibility testing. Characteristics of the participants are shown in Table 1. Of the 10 participating patients, most were male, with a median age of 63.5 years and had a higher professional education. Most participants lived with others and did not know which NYHA class they were in. Six patients had comorbidity such as diabetes, kidney insufficiency or asthma. Of the 5 participating nurses, all nurses were female with a median age of 51 years, and three of them were HF nurse specialists. The mean years of work experience was 13.9 (range 5–23 years).

**Table 1:** Characteristics of participants early-stage feasibility study

Characteristics of patients	
*N*	10
Age in years, median [IQR]	63.5 [20]
Female, n	4
Education, n	
Primary education Secondary education Secondary vocational education Higher professional education Academic education	0 1 2 5 2
Living status	
Alone With partner With partner & kids	1 5 4
NYHA functional class, n	
II IV Unknown	1 3 6
Characteristics HF nurses	
*N*	5
Age in years, median [IQR]	51 [17]
Female, *n*	5

Based on feedback on the paper-based version, several refinements were made to improve comprehensibility. We simplified the text, replaced difficult terminology with accessible language, and revised unclear or illogical response options. To improve accessibility, we added tailored multimedia materials to the digital intervention. This includes audio recordings of relaxation exercises, videos featuring HF nurses, and visuals that support the intervention’s topics. Based on user experiences with the digital intervention, further refinements were made to improve usability. We adjusted the layout, how users navigate, and how the system presents content to make the intervention more user-friendly for patients and nurses.

#### Acceptability

Patients stated that the use of positive and clear wording of the content of the intervention plays a key role in promoting patient motivation and engagement, ultimately supporting self-care behavior. Personal contact with a HF nurse was seen as valuable and could motivate participants to continue with the intervention. The modules were perceived as practical, inspiring, and easy to understand. Patients valued the basic component because it provided relevant, detailed information in an accessible way and particularly appreciated the small, manageable text sections. The first component was considered useful and applicable to daily life, partly because of the overview of HF symptoms. Patients welcomed the exercises in this module and found the instructions easy and similar to exercises in cardiac rehabilitation. In the second component, patients more frequently noted that the self-assessment included many questions, although this was not necessarily perceived as burdensome. They valued the structured step-by-step action plan that they could revisit in the third component. There was variation regarding which components patients found relevant. Some patients with longstanding HF felt that the basic component would be most suitable for patients who had recently been diagnosed with HF. HF nurses believed that the structural and practical support will improve patients’ self-monitoring and symptom management skills, which are often overlooked in their clinical practice.

*I think it’s a good package overall. I think if I were to receive this from the heart failure clinic*,* I’d be a satisfied patient. I hope to experience it in my lifetime*,* if it’s launched. (P9*,* 66 y old male patient)*

*I really enjoyed reading it. I also liked that I think it’s easy for patients to read*,* because the sentences are nice and short. It’s clear*,* doesn’t contain too much nonsense*,* and things like that. (P1*,* 66 years old male patient)*

*…Self-management or rather*,* the idea of self-management tends to get overlooked. Given the limited consultation hours*,* you have and the time allotted for each appointment. …There’s something to be gained there for patients who rely on informal caregivers*,* and for the caregivers themselves to find some peace of mind. Probably because her partner finds peace*,* and because they might both come to understand just how serious the condition is or be able to create a sense of reassurance at home*,* so to speak. (N2*,* 23 years of work experience)*

#### Demand

Patients reported that the intervention was helpful in several ways. They appreciated its personalized approach by providing insight into their individual symptom patterns, clarified their internal experiences, and stimulated them to reflect on their symptoms and behavior. Patients stated that they would recommend the intervention to peers, citing its potential to improve understanding of managing their HF. Regarding the first component, opinions differed on whether the exercises helped patients identify and recognize their symptoms. Some patients perceived the exercises as beneficial, whereas others remained uncertain. Patients indicated that repeated practice would likely be necessary to become familiar with the exercises. They emphasized that successful engagement with the intervention requires users to recognize the importance of their own role in symptom management. Also, users need to remain receptive to the intervention and willing to engage with the included exercises. Some patients noted that motivation may need to be strengthened through external support, such as; social influence, shared experiences of peers, or personal contact. The symptom management counselling component was experienced as both valuable and challenging. Patients felt that self-assessing encouraged them to take more ownership of their problems and care process. However, patients questioned whether all individuals can critically reflect on their own behavior. Nurses perceived that the intervention encouraged patients to explore alternative strategies to manage symptoms and take greater ownership of their care.

*The layered approach where if there’s a symptom*,* you can go into it more specifically. I really like that*,* because then you’re really targeting the specific intervention for that person or that patient*,* rather than just working with generalities*,* because I think that’s one of the main reasons why so many things don’t work*,* because it’s just not quite right for me. (P1*,* 66 years old male patient)*

*You must be open to it*,* like I said*,* … you must be willing to go along with it*,* right? So that it contributes to any potential improvement… If that’s not something you’re into as a patient*,* in my experience*,* well*,* then you’re not going to give it 100% … So that makes it difficult. (P4*,* 61 years old male patient)*

*Don’t be afraid to try other things to deal with a symptom so it bothers you less. That’s been my experience. I’m not saying it’s always a good idea*,* but don’t be afraid to push past that edge and see what it’s like. How it feels. Just let yourself feel overwhelmed or tired once*,* then you’ll know. You must experience that for yourself. And sometimes it’s not a bad thing to go over that edge once*,* if you come back down again. And that’s*,* of course*,* what people need to learn too. (P3*,* 70 years old female patient)*

*I was really excited about the whole platform…this is a way for patients to take matters into their own hands. And with a little encouragement from the nurse…I think that’s really your role as a nurse*,* to act as a coach*,* but also to provide real support. And you don’t always have to come up with a solution. Just giving a little recognition*,* acknowledging that what he/she did was good. (N3*,* 6 years of work experience)*

#### Implementation

Nurses valued the intervention for clinical practice because outpatient HF care currently underemphasizes self-management. One perceived advantage of the intervention was that follow-up visits may become less necessary, as the intervention itself already provides patients with feedback and support. Nurses initially felt overwhelmed by the number of actions necessary to use the intervention. Such as reading the answers of patients, giving feedback, and recording study-related activities in the intervention. However, they reported that this feeling improved with experience, as repeated use of the platform increased their speed and efficiency. One nurse also noted that healthcare professionals should complete the intervention themselves to fully understand the patient experience. Although this requires time investment, this could improve efficiency in the longer term. Also, nurses highlighted the importance of organizational support for successful implementation of the intervention within organizations. Some questioned whether healthcare organizations using telemonitoring systems would adopt an additional digital application.

*I think it saves time*,* because I wouldn’t have to cover that part during my outpatient appointment anymore. Or just very briefly*,* since it’s part of a nurse practitioner’s role anyway. But then you can do it more quickly. You can do a very brief evaluation. You no longer must explain to someone what self-care management is or what we expect from it*,* because they’ve already gone through that process. (N2*,* 23 years of work experience)*

*It is quite a lot*,* I’d say*,* just to get through it easily. And you’ll get used to it if you do it more often*,* but for the beginning*,* it is quite a lot just to figure out. (N1*,* 5 years of work experience)*

*Maybe you should pass that along as a tip: It’s another small investment but take a moment to read through the information patients actually receive*,* because that might make it easier to give feedback to them if you know they’ve already gone over it all. (N3*,* 6 years of work experience)*

#### Practicality

Patients provided constructive suggestions for improving wording to avoid psychological distance and address them directly. They preferred the use of “you” and “your,” as this increased perceived personal relevance and engaging. Patients also recommended using concrete sentences and providing alerts for difficult terminology when referred to external websites. Overall, patients perceived the intervention as detailed but time demanding. However, participants did not find the required time burdensome because they appreciated completing the intervention in multiple sessions rather than all at once. Participants noted that some users might lack the patience to complete longer exercises, although patience likely varies substantially between individuals. For example, the symptom list was described as extensive; however, patients considered this breadth necessary to ensure the list was comprehensive and more likely to feel personally relevant than a shorter version. They emphasized that adequate time allocation is required to complete the intervention, highlighting scheduling the intervention in daily life as an important practical consideration. Nurses indicated that they appreciated the availability of feedback templates, as providing individualized feedback without such support would require substantially more time. One nurse also provided constructive suggestions for improving the templates, noting that some example responses were perceived as overly vague or somewhat patronizing.

*Sometimes it says things like*,* ‘Fortunately*,* you can do a lot yourself.’ In those cases*,* you really feel directly addressed. But at other times it talks about ‘people’ this and ‘people’ that. Then I think: you are using this intervention because it concerns you personally. Of course*,* not everything will apply to everyone*,* but I would prefer the text to address the user directly throughout. As it is now*,* it sometimes feels a bit impersonal as if it is talking over your head.(P2*,* 47 years old female patient)*

*I mean*,* if I hadn’t had it*,* it would have taken me even longer*,* let me put it that way. Then I would be thinking*,* like*,* what should I write? And now I was like*,* oh yes*,* there*,* there*,* I start with that sentence and then*,* oh yes… so I really liked them a lot. I think you should keep that in there. (N3*,* 6 years of work experience)*

#### Integration

HF nurses emphasized the importance of communicating the benefits and added value of the STAR intervention to stakeholders within HF clinics. They also recommended providing guidance on integrating the intervention into existing care pathways. One nurse expected the intervention to fit within her current workflow, available time, and existing patient support practices to implement the STAR intervention feasibly and efficiently. Nurses anticipated that STAR would help patients monitor and manage symptoms better, reducing unplanned consultations. They also indicated that the intervention aligns with ongoing transitions toward hybrid and online models of healthcare delivery. Thus, it fits within contemporary clinical practice. Nurses expressed different perspectives on how the intervention should be integrated into routine patient care. Some preferred supporting their own patients because they knew the patients and their situations. Others argued that care should remain transferable among healthcare professionals. They suggested that assigning patients to individual nurses during a trial may be impractical. Differences were also reported about the preferred timing of feedback during the intervention. Some nurses preferred providing feedback between daily work tasks; others preferred scheduling feedback at fixed moments in their calendars. One nurse described that online platforms add workload to existing clinical responsibilities and questioned how to incorporate sufficient time for online patient monitoring into routine care. Nurses emphasized that healthcare providers must carefully consider when and to whom to introduce the intervention to patients. Some nurses believe that offering the intervention shortly after an HF diagnosis may overwhelm patients. Also, they argue that patients with advanced HF require more intense instructions on using the intervention.

*I think it will reduce the need for unnecessary phone consultations. People are anxious or afraid at home*,* or they’re not quite sure what to do about their symptoms. And if we can reassure them for just a moment*,* they’ll learn to recognize those feelings better*,* and they’ll find the peace of mind that comes from knowing it all resolves itself with a breathing exercise. So yes*,* that also reduces the burden on phone consultations and healthcare utilization. (N2*,* 23 years of work experience)*

*I’m just being personal here*,* but …I’m just not really into all that platform stuff because I always think*,* “Oh*,* that’s going to be a lot of extra work.” Like*,* “Oh*,* do we really have to monitor all those extra patients online too? … I don’t know where I’m supposed to find the time. N1*,* 5 years of work experience)*

*On the one hand*,* I think*,* yes*,* those vulnerable*,* fragile advanced HF patients…*,* but on the other hand*,* there are also features on that platform that can help them. They just need to be clearly instructed that this platform isn’t the place to turn to when things are really bad. So*,* I think there are useful things on there for those patients as well. (N3*,* 6 years of work experience)*

#### Usability

Patients and nurses perceived the STAR intervention as user-friendly and structured in a logical sequence of modules. The user-friendliness and the clear and basic layout were viewed positively. Login was easy without technical issues. Patients easily accessed the components and navigated through the intervention. Patients indicated that there should be a mix of text, icons, audio, and video. Nurses mostly provided suggestions related to practical aspects such as the number of clicks needed. Also, nurses noted that the more they used the intervention, the more comfortable they became with navigating it and knowing where to provide feedback to patients.

*It is user-friendly. You can stop in between sessions*,* see where you left off*,* and review your previous answers…I think that is especially important for someone who has just been diagnosed with HF*,* because you can go back and reread the information. It is not only about a doctor telling you what to do; you also need to adapt your own life. This intervention can support you in doing that. (P10*,* 48 years old male patient)*

*I also think that the more often you use this…the more you begin to recognize the moment when someone has truly completed a step and is ready to move on to the next component and start acting. With only a single test session*,* you simply do not develop that sense yet (N3*,* 6 years of work experience)*

## Discussion

Investing time in preparation of behavioral interventions is important to strengthen methodological rigor and to reduce research waste [35]. However, guidance on how to develop a conceptual model within MOST remains limited, and only a few studies describe how activities in the preparation phase contribute to the optimization process [23, 36]. The present study contributes to this gap by providing a structured and transparent description of these processes.

An interesting finding in this first phase of MOST was the role of mindfulness. Mindfulness-based interventions are increasingly applied in chronic disease management and have shown potential to improve symptom awareness and emotional regulation [6]. However, the experts in our study advised against incorporating a full mindfulness program into our STAR intervention. They viewed a full mindfulness intervention as a therapeutic approach requiring intensive guidance and expertise, which also does not align with the aim of a brief, scalable self-care intervention. This highlights the need to balance theory with feasibility and implementation of the intervention. Therefore, integrating selected mindfulness-inspired elements may fit the context better than a full mindfulness program. This approach can still support patients’ awareness of bodily sensations and will be tested in the subsequent optimization trial [37].

Furthermore, patients perceived the reflective exercises both as a strength and a challenge of the intervention. Several participants reported that reflecting helped them recognize symptoms. Others found these exercises cognitively demanding. This suggests that reflection may support self-care by fostering insight and ownership, but that its benefits depend on patients’ cognitive capacities, motivation, literacy, and personal circumstances [4]. This is something we need to keep in mind for further evaluation of the STAR intervention. Reflective opportunities must be balanced with the flexibility to accommodate individual preferences and abilities [22].

Another notable finding was the importance to patients for personal contact with HF nurses. Although participants found the intervention informative and supportive, they emphasized that nurses could motivate them to continue using it. These findings suggest that digital self-care interventions may be more effective when embedded within clinical care than when offered as standalone tools [22, 38]. Nurses can provide reassurance, accountability, and tailored support to help patients engage with these interventions over time [22, 32, 39]. At the same time, this finding highlights an important challenge. The STAR intervention aims to strengthen patients’ ability to recognize symptoms, interpret their meaning, and respond appropriately rather than increase their reliance on healthcare professionals. As patients develop these self-care skills with the STAR intervention, the need for intensive nurse support may decrease. Thus, the STAR intervention can support greater patient autonomy and reduce the demand for routine professional support. In the subsequent optimization trial, we will optimize the STAR intervention by identifying which components require personal nurse support. We will examine whether nurse support enhances patient engagement, but also the minimum level of support required to maintain patients’ confidence and self-care behavior.

From the nurses’ perspective, the STAR intervention addressed a gap in routine HF care by providing structured support for self-care management. However, nurses feared that the program would increase their workload. These concerns regarding workload and organizational support, show that success depends on more than the intervention itself. Nurses anticipated that the STAR intervention would seem less complex as they gained experience. This suggests that implementation barriers may reflect an early learning period rather than permanent obstacles [22]. To address these barriers, we need to focus on training, support, and integration within existing clinical workflows in the optimization trial [40].

### Future research

Feedback from both patients and nurses is valuable as it allows us to refine content, delivery, and implementation procedures to better support engagement of users and fit various contexts. The feasibility findings offer concrete and actionable input for us to further refine the STAR intervention. The next step is an optimization trial, to determine which intervention components and component levels of the STAR intervention contribute most to patient outcomes. Findings from this trial will be used to optimize the STAR intervention before evaluating its effectiveness.

### Strengths and limitations

A key strength of this study is the transparent and structured description of the activities involved in the preparation phase of MOST. By systematically outlining how the conceptual model, candidate components, and component levels were developed, this study contributes to the methodological literature on complex intervention design. Also, this paper may support other researchers in applying MOST. Another strength is the involvement of a diverse group of stakeholders, including researchers, healthcare professionals, and patients with HF, that improves relevance and acceptability of our intervention [41].

This study also has limitations. First, the intervention was initially reviewed in a paper-based format, which may have made it difficult for participants to fully understand how the digital intervention would work in practice. Some aspects are easier to experience than to imagine; however, this paper-based review enabled early refinement of the digital intervention.

Second, the conceptual model reflects a set of mechanisms about how we expect the intervention is going to work. These mechanisms have not yet been tested and may simplify the complexity of patient behavior and contextual factors influencing self-care.

Third, although the STAR intervention targets patients with stable HF (NYHA class II/III), the study also included nurses specialized in advanced HF and patients with NYHA class IV. Consequently, the study participants in the feasibility study did not fully correspond to the intended target population of the STAR intervention. Patients with NYHA class IV often experience greater symptom severity, reduced physical functioning, more complex care needs, and more motivation to engage in self-care. These factors may affect their perceptions of and how they engage with the intervention. Similarly, nurses specialized in advanced HF may hold perspectives that differ from nurses treating patients with stable HF. Thus, the feasibility outcomes may either underestimate or overestimate the intervention’s suitability for the intended population. This broader inclusion of participants was primarily driven by recruitment challenges among both patients and nurses. Additionally, although NYHA classification was not available for all participating patients, it can be assumed that participants had at least NYHA II, as the recruitment flyer specified that patients needed to be symptomatic to participate in the feasibility study. However, in future studies we could estimate someone’s NYHA functional class in case of missing data [42].

### Implications for nursing practice

Our findings have several implications for nursing practice. Nurses play an important role in supporting patients in recognize and respond to symptoms. This suggests that support is particularly important for fluctuating daily symptoms that do not require immediate medical attention. Nurses can use the STAR intervention to complement existing HF care. Patients take the lead in their own symptom monitoring and management, and nurses provide support where needed. Through the intervention, the patient can learn a strategy for recognizing symptoms early and taking timely appropriate actions. Nurses can use the patient’s input during consultations and use these insights to guide and support patients in their self-care. However, the optimization trial must determine the optimal extent, form, and timing of support and guidance. In this optimization trial, we will also investigate how nurses can integrate the STAR intervention into current work processes.

### Conclusion

We developed a conceptual model, identified candidate components and optimization criterion, and tested the intervention in a feasibility study. By linking intervention components to underlying mechanisms and outcomes, our conceptual model provides a foundation for optimization. The STAR intervention is feasible and acceptable to both patients with HF and HF nurses. The next step will be refining the STAR intervention before we evaluate the candidate components in a subsequent optimization trial, which is essential to determine which components contribute most to improving patient outcomes.

## Supplementary Information

Below is the link to the electronic supplementary material.

Supplementary Material 1

## Data Availability

No datasets were generated or analysed during the current study.

## Abbreviations

HF: Heart failure
MOST: Multiphase Optimization Strategy
RCT: Randomized controlled trial
STAR: SympTom mAnagement and self-caRe
NYHA: New York Heart Association
