# Supplementary material for: Developing a digital heart failure symptom management intervention using the preparation phase of the Multiphase Optimization StraTegy
Source: BMC Nurs. 2026 Oct 22;25:1012. doi: 10.1186/s12912-026-05571-2 (PMC13693238; doi:10.1186/s12912-026-05571-2)
Supplement: Supplementary file 1 [file 12912_2026_5571_MOESM1_ESM.pdf]

Appendix

**Multiphase Optimization STrategy (MOST) PREParation REPorting (PREP-REP) Checklist**<br>This checklist should be used to guide the reporting of the process and outcomes of the MOST preparation phase in publications, presentations, and other research products.<br><br>**1 Introduce The MOST Framework**<br>1.1 Identify the use of the multiphase optimization strategy (MOST) with appropriate citation(s)<br>1.2 Label aspects of current or prior work that the research team considers preparation for an optimization phase as the preparation phase<br><br>**2 Describe the Research Series**<br>2.1 Provide context for the current work in the introduction or background and clearly label prior, ongoing, or planned work as preparation where appropriate<br>2.2 Describe the trajectory of the research program leading up to the current work<br>2.2.a. Provide citations for any prior or associated work whether it was completed by the research team or by others (e.g., literature reviews, formative research, pilot studies, trials)<br>2.2.b. Connect the trajectory of the research program to the MOST phase(s) by labeling prior and current work with the appropriate phase (e.g., preparation phase, optimization phase) including any iterative movement between phases<br>2.2.c If space is available, provide example(s) of how prior work was translated into elements of the conceptual model and/or the optimization objective<br><br>**3 Clearly Define the Conceptual Model**<br>3.1 Use the term conceptual model to identify the theory or framework guiding the selection of the components, mediators, and outcomes for optimization<br>3.2 Provide a detailed description of the conceptual model including a figure or diagram<br>3.2.a Identify and describe the intervention components and levels<br>3.2.b. Identify primary (and secondary) outcomes being targeted by the intervention components including descriptions of assessment tools<br>3.2.c Describe how each intervention component is purported to affect each outcome including descriptions of how relevant mediators will be assessed<br><br>**4 Optimization Objective**<br>4.1 Use the term optimization objective to identify the factors that will guide the selection of intervention components after the optimization trial<br>4.2 Provide an operational definition of the optimization objective<br>4.2.a Include specific criteria, if known, for determining which components will be retained in optimized intervention such as benchmarks related to effectiveness, affordability, scalability, and/or efficiency.<br>4.3 Describe the rationale for the selection of the optimization objective	Author responses<br><br>1.1. Description of MOST in sub-section in introduction<br>1.2. The entire paper is labeled as preparation phase.<br><br>2.1. The context for current work is described in the STAR intervention described in the introduction.<br>2.2.a. References 4- 6, 14, 31-33<br>2.2.b. The entire paper is labeled as the preparation phase.<br>2.2.c. Conceptual model is described in Activity 1 and Figure 2.<br><br>3.1. Conceptual model is described in Activity 1 and Figure 2.<br>3.2.a. Intervention component en levels are described Activity 2 and 3 and Figure 3.<br>3.2.b. Proximal and distal outcomes are described in Activity 1 results section<br>3.2.c. Described in Activity 1 results section<br><br>4.1. Optimization objective is described in Activity 2/3 method section<br>4.2. Our optimization objective is described in Activity 2/3 method section<br>4.3. Rationale for the optimization objective is discussed in Activity 2/3 results section
